# Role of the IL-6 Gene in the Etiopathogenesis of Idiopathic Scoliosis

**DOI:** 10.1155/2015/621893

**Published:** 2015-06-23

**Authors:** Svetla Nikolova, Milka Dikova, Dobrin Dikov, Assen Djerov, Gyulnas Dzhebir, Ventseslav Atanasov, Alexey Savov, Ivo Kremensky

**Affiliations:** ^1^National Genetic Laboratory, Medical University-Sofia, 2 Zdrave Street, 1431 Sofia, Bulgaria; ^2^University Orthopedic Hospital “Prof. Boycho Boychev”, 56 Nikola Petkov Boulevard, 1614 Sofia, Bulgaria; ^3^Molecular Medicine Center, Medical University-Sofia, 2 Zdrave Street, 1431 Sofia, Bulgaria

## Abstract

Scoliotic human nuclei pulposi can respond to exogenous proinflammatory stimuli by secreting increased amounts of interleukin-6 (IL-6). The G/C polymorphism of the promoter region of *IL-6* gene influences levels and functional activity of the IL-6 protein. We conducted a case-control study of eighty patients with idiopathic scoliosis (IS) and one hundred sixty healthy unrelated gender-matched controls trying to investigate the association between IS and the *IL-6* promoter polymorphism at -174 position (rs1800795 G/C) in Bulgarian population. Molecular detection of the *IL-6* genotypes was performed by amplification followed by restriction technology. The statistical analysis was performed by Pearson's chi-squared test. Our case-control study revealed a statistically significant association between the *IL-6* (-174 G/C) functional polymorphism and susceptibility to IS. In addition, a significant association between the *IL-6* (-174 G/C) polymorphism and curve severity was detected. *IL-6* gene could be considered as susceptibility and modifying factor of idiopathic scoliosis. The identification of molecular markers with diagnostic and prognostic value could be useful for early detection of children at risk for the development of scoliosis and for prognosis of the risk for a rapid deformity progression. That would facilitate the therapy decisions and early stage treatment of the patient with the least invasive procedures.

## 1. Introduction

Interleukin-6 (IL-6) is involved in the activation of the immune system, in regenerative processes, in the regulation of metabolism, in the maintenance of bone homeostasis, and in many neural functions [1].

It is now well established that degeneration of the intervertebral discs (IVDs) is a part of very complex cascade of events initially triggered by a rise in levels and activities of inflammatory cytokines. Among those closely linked to disc tissue breakdown are tumor necrosis factor alpha (TNF-*α*), interleukin-1*α* (IL-1*α*), interleukin-1*β* (IL-1*β*), IL-6, interleukin-8 (IL-8), and interleukin-17 (IL-17). In addition, other metabolites linked to the inflammatory response including prostanoids, thromboxanes, and nitric oxide are present in degenerating discal tissues. These agents enhance the expression of the matrix degrading enzymes like matrix metalloproteinases. The elevation in the activities of these enzymes promotes the breakdown of aggrecan, the major proteoglycan of the extracellular matrix of both the nucleus pulposus and the annulus fibrosus. Aside from proteoglycans, these agents cleave fibrillar proteins of the disc extracellular matrix. In addition to promoting these catabolic processes, the cytokines influence cell metabolism in a number of ways. First, they suppress processes that mediate matrix repair. Second, the cytokines enhance intracellular events that significantly degrade nucleus pulposus and annulus fibrosus function. Third, the cytokines promote cellular events that cause death by apoptosis [2].

The resulting imbalance in catabolic and anabolic responses leads to the degeneration of IVD tissues, as well as disc herniation and radicular pain. The release of chemokines from degenerating discs promotes the infiltration and activation of immune cells, further amplifying the inflammatory cascade. Recent evidence suggests that inflammation is one of the key factors in the etiology of intervertebral disc disease [3].

Burke et al. [4] cultivated human disc tissue obtained from patients undergoing surgery for scoliosis and then analyzed the medium for the production of a range of proinflammatory mediators. They concluded that both scoliotic and degenerate human nucleus pulposus can respond to an exogenous proinflammatory stimulus by secreting increased amounts of IL-6, IL-8, and PGE2, but not TNF-alpha, and that degenerate disc tissue is more sensitive to a proinflammatory stimulus than its scoliotic counterpart.

The G/C polymorphism of the promoter region of* IL-6* gene influences levels and functional activity of the IL-6 protein. The transfection of* IL-6*-174C alleles into HeLa cells in vitro has resulted in a decrease in IL-6 production compared to that of* IL-6*-174G allele [5].* IL-6 *(-174 C/G) is involved in the pathogenesis of some autoimmune disorders. Hristova et al. [6] reported a statistically significant association between* IL-6*-174G allele and systemic lupus erythematosus in Bulgarian population.

A case-control study conducted by Aulisa et al. in 2007 was the first one to investigate the role of the* IL-6* (-174 C/G) functional polymorphism in the pathogenesis of idiopathic scoliosis (IS). IS is a complex developmental syndrome that manifests primarily as an abnormal structural curvature of the spine that involves changes in the frontal, sagittal, and transversal planes. Eighty percent of all spinal curvatures are clinically classified as idiopathic, making IS the most common form of spinal deformity (OMIM 181800). The current consensus on IS maintains that it has a multifactorial etiology with genetic predisposing factors. Aulisa et al. [7] concluded that the* IL-6* (-174 C/G) promoter polymorphism constitutes important factor for the genetic predisposition to scoliosis.

No significant differences in the genotype and allele frequencies of the* IL-6* (-174 C/G) polymorphism were reported between the cases and controls in Hungarian population sample [5] and Chinese Han population [8]. Other polymorphisms in* IL-6* were associated with bone mineral density in Korean girls with adolescent idiopathic scoliosis (AIS) [9].

In the present study, the association of the* IL-6* (-174 C/G) functional polymorphism with IS was investigated among Bulgarian patients.

## 2. Materials and Methods

In this study, patients with IS (*n* = 80) and healthy unrelated gender-matched controls (*n* = 160) were included. All participants in the study were informed about its purpose and were included only after the subjects/families signed their informed consent. Peripheral blood samples were obtained from patients and control subjects. The study protocol was approved by the Ethics Committee of the Medical University-Sofia (number 2987/2012).

### 2.1. Patients

Patients with IS were recruited with the help of orthopaedic surgeons from University Orthopedic Hospital “Prof. Boycho Boychev.” The IS diagnosis was confirmed clinically and radiologically. Scoliosis as a phenotypic characteristic like Marfan's syndrome was excluded. The curves were measured by the Cobb method. The mean value of Cobb angles was 54.6 ± 23.2. The mean age at the beginning of the disease was 11.2 ± 3.1 years.

The patients were grouped by 4 indices: (1) age of the onset of the disease: infantile, juvenile, or adolescent idiopathic scoliosis; (2) familial history: familial or sporadic cases; (3) Cobb's angle: in surgical and nonsurgical treatment; (4) gender: males and females.

IS was observed in three age groups: infantile, from one to three years of age (*n* = 3), juvenile, from greater than three years of age to nine years of age (*n* = 16), and adolescent (AIS), from ten to sixteen years of age (*n* = 61). Patients with positive familial history (*n* = 22) and sporadic cases (*n* = 58) were included. The Cobb angle under consideration was either the final Cobb angle, defined as the curve angle in skeletally mature patients (*n* = 35), or Cobb angle, measured in the last followup of those patients who were skeletally immature (*n* = 45). Patients were assigned to two treatment groups: one surgical (*n* = 62) and one nonsurgical (*n* = 18). In this study, male (*n* = 15) and female (*n* = 65) patients were included.

### 2.2. Controls

The control group including healthy subjects without clinical signs of IS was recruited from a pool of unrelated gender-matched volunteers from other units and clinics of Tokuda Hospital Sofia, National Genetic Laboratory, hospital staff members, and students. The controls were selected among adult patients with skeletal maturity with negative family history of IS. Radiological examination was not performed in the control group.

### 2.3. Genotyping

Genomic DNA was extracted from the peripheral blood leucocytes using magnetic bead technology (chemagic DNA Blood Kit special, Chemagen) on automated high throughput nucleic acid isolation platform (chemagic Magnetic Separation Module I, Chemagen).

The polymorphic region of the* IL-6* gene was amplified by polymerase chain reaction (PCR). The primer set [5] is listed in Table 1.

The PCR was carried out in a reaction mix of 20 *μ*L containing 100 ng DNA and 10x Prime Taq buffer (Genet Bio), 10 mM dNTPs Mixture (Genet Bio), 20 pmol Forward and Reverse primers (AlphaDNA), and 0.1 U Prime Taq DNA Polymerase (Genet Bio). PCR amplification was performed in an AB 2720 Thermocycler (Life Technologies) with an initial denaturation at 94°C for five minutes and a final extension of seven minutes at 72°C. The following thermal cycle was repeated 30 times: denaturation at 94°C for 30 seconds, annealing for 30 seconds at temperature presented in Table 2, and extension at 72°C for 30 seconds.

The PCR product was cleaved with SfaNI restriction endonuclease (New England Biolabs), according to the manufacturer's instructions, and the restriction fragments were separated on agarose 3% gel in VG-SYS Horizontal Electrophoresis System (Biochrom). The lengths of the fragments representing the genotypes of* IL-6* are presented in Table 2.

### 2.4. Statistical Analysis

The statistical analysis was performed by Pearson's chi-squared test to make genotype and allele comparisons between cases and controls as well as test for Hardy-Weinberg equilibrium. A value of *p* < 0.05 was considered to be statistically significant for comparison between data sets. Phi and Cramer's *V* were calculated. Odds ratios (ORs) of major* versus* minor homozygote genotypes and alleles were calculated with 95% confidence interval (95% CI). Statistical analysis was conducted with the SPSS 19.0 software package for Windows.

## 3. Results

This study examined the association between IS and the* IL-6* promoter polymorphism at -174 position (rs1800795 G/C).

The overall frequency of the GG genotype of* IL-6 *(-174 C/G) in the patients with IS was significantly higher than that in the controls (52.5% versus 35.0%; *p* = 0.009) and the frequency of the G allele of* IL-6* in the patients with IS was also higher than that in the controls (70.6% versus 54.4%; *p* = 0.0006). In conclusion, the homozygous GG genotype of* IL-6* was associated with a higher risk of scoliosis (GG versus CC, OR: 3.5; 95% CI: 1.54–7.98) and the presence of the G allele (G versus C, OR: 2.02; 95% CI: 1.35–3.03) could be considered as susceptibility factor to IS.

The genotype/allele frequencies of the case/control group are summarised in Table 3.

In the subgroup of AIS patients (*n* = 61), the frequencies of the GG genotype and the G alleleof* IL-6* were significantly higher than those in the controls (*p* < 0.05) and elevated ORs were observed (GG versus CC, OR = 3.75; 95% CI: 1.52–9.27 and G versus C, OR = 2.3; 95% CI: 1.28–4.16).

In the subgroup of the familial cases (*n* = 22), the frequencies of the GG genotype and the G allele of* IL-6* were significantly higher than those in the controls (*p* < 0.05). The GG genotype and the G allele showed increased ORs (GG versus CC, OR = 10.5; 95% CI: 1.33–83.03 and G versus C, OR: 3.26; 95% CI: 1.52–7.01).

In the surgical treatment group (*n* = 62) where Cobb angle >40°, the frequencies of the GG genotype and the G allele of* IL-6* were significantly higher than those in the controls (*p* < 0.05) and increased ORs were observed (GG versus CC, OR = 4.25; 95% CI: 1.63–11.05 and G versus C, OR = 2.3; 95% CI: 1.28–4.16, resp.).

In the group of male patients (*n* = 15), the frequency of the G allele of* IL-6* was significantly higher than that in the control male group (*n* = 30). The G allele showed increased OR (G versus C, OR: 3.27; 95% CI: 1.17–9.16).

In the group of female patients (*n* = 65), the frequencies of the GG genotype and the G allele of* IL-6* were significantly higher than those in the female controls (*n* = 130) and the GG genotype and the G allele of* IL-6* showed increased ORs (GG versus CC, OR = 3.02; 95% CI: 1.24–7.39 and G versus C, OR = 1.9; 95% CI: 1.06–3.38).

Odds ratios of genotypes and alleles in the subgroups are summarised in Table 4.

## 4. Discussion

In our study, the frequencies of the genotypes of* IL-6* showed statistically significant differences between cases and controls (*χ* ^2^ = 9.735; *p* = 0.008) and the GG genotype of* IL-6* could be considered as a predisposing factor for IS (GG versus CC, *p* = 0.002; OR = 3.5). The G allele was associated with higher risk for development of IS (G versus C, OR: 2.02; 95% CI: 1.35–3.03). In this way, a previously reported genetic association between* IL-6* (-174 G/C) and the predisposition to IS in Italian patients [7] was confirmed among Bulgarian patients.

In the subgroup of adolescent patients (*n* = 61), a strong association between the GG genotype and the G allele of* IL-6* and the clinical phenotype was observed (Table 4). Adolescent idiopathic scoliosis (AIS) is the most common spinal deformity and the most frequently studied idiopathic scoliosis [5, 7]. Our results revealed comparable associations between the* IL-6* genetic polymorphism and IS in the AIS group and in the total group of patients (Table 4). These results suggest that there is genetic predisposition for all forms of IS, but much larger case-control study will be necessary to examine the role of* IL-6* in the etiology and pathogenesis of infantile and juvenile scoliosis.

Predisposition for IS, like other examples of complex traits, does not have a specific assigned risk of heritability, but inheritance is based on multiple factors, potentially both genetic and environmental [10]. In the subgroup of the familial cases (*n* = 22) a significant association between* IL-6* and IS was found (Table 4). These results suggest a different model of inheritance for familial and sporadic cases with a higher impact of genetic factors on the etiology of the familial idiopathic scoliosis.

In the subgroup of surgical cases (*n* = 62) where Cobb angle >40°, a strongly significant association between* IL-6* and IS was detected (*χ* ^2^ = 10.467; *p* = 0.03). The results of the statistical analysis in this study indicate that the* IL-6 *(-174 C/G) polymorphism was significantly associated with curve severity (Table 4). On the basis of these results,* IL-6* could be considered as a gene with modifying effect on the pathological phenotype. Replication studies should be carried out to confirm this result.

Idiopathic scoliosis is more common in females than males. In the subgroup of female patients (*n* = 65) a statistically significant association between the* IL-6* promoter polymorphism and the clinical phenotype was observed (Table 4). In the small subgroup of male patients (*n* = 15), this association is less significant and our results suggest that there is genetic predisposition for IS in male and female patients but much larger case-control study will be needed to examine the role of* IL-6* in the etiology and pathogenesis of IS in males.

Our study used identical technical approaches compared to other studies [5, 7–9] on genetics of idiopathic scoliosis and the role of* IL-6* in the etiopathogenesis of the disease as amplification-restriction (PCR-RFLP) and statistical analysis of data with SPSS for Windows.

First, the observed differences in the results between the different population groups could be explained with different selection criteria for the samples (preanalytical phase), technical errors (analytical phase), and differences in the preferred statistical methods with or without corrections (postanalytical phase). Second, the genotype and allele frequencies could be different in the different population and even ethnical groups.

Ivanova et al. in 2001 presented for the first time HLA class I allele and haplotype frequencies at DNA level in the Bulgarian population. HLA class I profile of Bulgarians has been compared to other European and Mediterranean populations of common historical background in order to clarify more precisely the origin of our population. Genetic distances, phylogenetic trees, and correspondence analyses show that the Bulgarian population is more closely related to the Italian, the Mediterranean, the Armenian, and the Romanian population than to the other East and West European populations [11].

Our results were close to the results observed in the Italian population sample [7], but their study includes relatively small number of patients and it could explain the higher value of odds ratio compared to our study.

## 5. Conclusions

In conclusion, this case-control study revealed statistically significant association between the* IL-6* (-174 G/C) functional polymorphism and the susceptibility to IS and confirmed a previously reported genetic association between the* IL-6* -174G allele and predisposition to IS in Italian patients [7]. In addition, a significant association between the* IL-6* (-174 G/C) promoter polymorphism and curve severity was detected among Bulgarian patients.

Our results suggest that the identification of molecular markers with diagnostic and prognostic value could be useful for early detection of children at risk for the development of IS and for prognosis of the risk for a rapid deformity progression. That would facilitate the therapy decisions and early stage treatment of the patient with the least invasive procedures. An extended population-based case-control study is necessary to confirm the contribution of this polymorphic variant of* IL-6* to the development and progression of IS.

## Figures and Tables

**Table 1:** PCR primers.

Gene, polymorphism	Primers
*IL-6* *rs1800795 *	Forward: 5′-TGACTTCAGCTTTACTCTTTgT-3′
	Reverse: 5′-AAAGCAAAGACAGGCATAAA-3′

PCR indicates polymerase chain reaction; *IL-6*: interleukin-6.

**Table 2:** PCR-RFLP protocol.

Gene, polymorphism	Annealing temperature, °C	PCR product size, bp	Restriction enzyme	Restriction fragments, bp
*IL-6* *rs1800795 *	58	198	SfaNI	CC: 198 CG: 198 + 140 + 58 GG: 140 + 58

PCR indicates polymerase chain reaction; RFLP: restriction fragment length polymorphism; *IL-6*: interleukin-6; bp: base pair.

**Table 3:** Genotype and allele frequency distributions in patients (*n* = 80) with idiopathic scoliosis (IS) and healthy controls (*n* = 160).

Gene, polymorphism	Genotype, allele	Cases %	Controls %	*p* value
*IL-6* *rs1800795 *	GG	52.5	35.0	0.008
	CG	36.3	38.8	
	CC	11.3	26.3	
	G	70.6	54.4	0.0006
	C	29.4	45.6	

A value of *p* < 0.05 was considered to be statistically significant. *IL-6* indicates interleukin-6.

**Table 4:** Odds ratios of genotypes and alleles in different subgroups with idiopathic scoliosis (IS).

Subgroup	Genotype, allele	*p* value	OR [95% CI]
General	GG versus CC	0.002	3.5 [1.54–7.98]
	G versus C	0.0006	2.02 [1.35–3.03]

AIS (*n* = 61)	GG versus CC	0.003	3.75 [1.52–9.27]
	G versus C	0.005	2.3 [1.28–4.16]

Familial history of IS (*n* = 22)	GG versus CC	0.007	10.5 [1.33–83.03]
	G versus C	0.002	3.26 [1.52–7.01]

Cobb angle >40° (*n* = 62)	GG versus CC	0.002	4.25 [1.63–11.05]
	G versus C	0.005	2.3 [1.28–4.16]

Males (*n* = 15)	GG versus CC	0.1	7.27 [0.77–68.89]
	G versus C	0.02	3.27 [1.17–9.16]

Females (*n* = 65)	GG versus CC	0.01	3.02 [1.24–7.39]
	G versus C	0.03	1.9 [1.06–3.38]

A value of *p* < 0.05 was considered to be statistically significant. AIS indicates adolescent idiopathic scoliosis; OR: odds ratio; CI: confidence interval.
